# A retrospective study on molar furcation assessment via clinical detection, intraoral radiography and cone beam computed tomography

**DOI:** 10.1186/s12903-018-0544-0

**Published:** 2018-05-03

**Authors:** Wenjian Zhang, Keagan Foss, Bing-Yan Wang

**Affiliations:** 1 Department of Diagnostic & Biomedical Sciences, University of Texas School of Dentistry at Houston, 7500 Cambridge Street, Houston, TX 77054 USA; 2 University of Texas School of Dentistry at Houston, 7500 Cambridge Street, Houston, TX 77054 USA; 3 Department of Periodontics & Dental Hygiene, University of Texas School of Dentistry at Houston, 7500 Cambridge Street, Houston, TX 77054 USA

**Keywords:** Furcation involvement, Clinical detection, Intraoral radiography, CBCT

## Abstract

**Background:**

Accurate determination of bone loss at the molar furcation region by clinical detection and intraoral radiograph is challenging in many instances. Cone beam computed tomography (CBCT) is expected to open a new horizon in periodontal assessment. The purpose of this study was to compare and correlate accuracy of molar furcation assessment via clinical detection, intraoral radiography and CBCT images.

**Methods:**

Eighty-three patients with chronic periodontitis who had existing CBCT scans were included. Furcation involvement was assessed on maxillary and mandibular first molars. Periodontal charts (modified Glickman’s classification), intraoral (periapical and/or bitewing) radiographs (recorded as presence or absence) and axial CBCT reconstructions were used to evaluate furcation involvement on buccal and palatal/lingual sites. The correlation of furcation assessment by the three methods was evaluated by Pearson analysis.

**Results:**

There were significant correlations (*p* < 0.05) between clinical detection and intraoral radiography, clinical detection and CBCT, as well as intraoral radiography and CBCT at all the measured sites (*r* values range between 0.230 to 0.644). CBCT generally exhibited higher correlation with clinical detection relative to intraoral radiography, especially at distal palatal side of maxillary first molar (*p* < 0.05). In addition, CBCT provided more accurate assessment, with bone loss measurement up to 2 decimals in millimeters, whereas clinical detection had 3 classes and the intraoral radiographs usually only detected the presence of furcation involvement in Glickman Class 2 and 3.

**Conclusions:**

This study validates that CBCT is a valuable tool in molar furcation assessment in addition to clinical detection and intraoral radiography.

## Background

Furcation involvement (FI) refers to the condition when periodontal disease has caused bone resorption into the bifurcation or trifurcation of a multi-rooted tooth [1]. Dentists commonly encounter the difficulty of accurately assessing molars with FI, due to limited physical access, morphological variations and measurement errors [2–4]. Any discrepancies between pre-and intra-surgical findings of FI may lead to alterations of surgical treatment plan [5] and unanticipated treatment costs (financially and temporally) [6]. Therefore, management of FI has presented as one of the greatest challenges to the success of periodontal therapy [7].

Traditionally, FI is assessed with a combination of clinical detection and intraoral radiographs [8]. Clinically, FI is evaluated with a Nabers probe, and categorized according to Glickman’s or Hamp’s classification system based on horizontal bone loss at the furcation area [9, 10]. However, the accuracy of clinical detection largely depends on operator technique, and many times, the measurement is reflective of penetration depth into the inflamed connective tissue, instead of the actual depth of the inter-radicular bony defect [11]. In addition, factors such as tooth position, inclination, root morphology, length of root trunk, degree of root separation and configuration of residual inter-radicular bone, all affect accuracy of clinical furcation assessment [12, 13]. Periapical (PA) or bitewing (BW) radiographs are commonly used intraoral projections to supplement clinical detection for furcation assessment [8, 14]. These 2D imaging are generally considered to have low sensitivity but high specificity for furcation detection, mainly due to inherent shortcomings of 2D projections, such as superimposition and angulation problems [15]. Detectability of early FI by intraoral radiographs is especially limited and inconsistent [16].

Cone beam computed tomography (CBCT) is capable of generating accurate and reliable submillimeter-resolution images in all spatial dimensions, with cost and absorbed doses much lower than conventional CT [17, 18]. The applications of CBCT in dentistry are increasing rapidly, including in periodontology [19, 20]. CBCT is expected to reveal marginal bone contours as well as infrabony and furcation defects [21], therefore plays a role in the assessment and treatment planning of molars with FI. Currently, there are limited studies comparing diagnostic accuracy of FI by clinical detection, intraoral radiography and CBCT [12, 22, 23]. The aim of the present study was to compare and correlate assessment of molar FI via these three methods, to help further develop evidence on the applicability of CBCT in molar FI assessment.

## Methods

### Subjects

An Institutional Review Board (IRB) approval was granted prior to the start of the study (HSC-DB-17-0370). The patients who visited the University of Texas School of Dentistry at Houston dental clinic from 2012 to 2016 were retrospectively screened according to the selection criteria. The inclusion criteria were: 1) subject was diagnosed as having generalized moderate or severe chronic periodontitis; 2) subject had comprehensive periodontal examination and information had been stored in the school’s Electronic Health Record (EHR); 3) subject had diagnostic quality periapical and/or bitewing radiographs covering posterior dentition; 4) subject had diagnostic quality CBCT scan with coverage of entire maxilla and mandible. Majority of the patients had CBCT scans for implant treatment planning purpose, and the time interval between periodontal clinical exam and CBCT scan was less than 3 months. All of the patients who met the inclusion criteria were included in the study, and their first molars of maxilla and mandible bilaterally were assessed according to the following methods.

### Intraoral radiographic acquisition

All the intraoral radiographs were acquired with Focus wall-mounted unit (Instrumentarium Dental, Charlotte, NC, USA). The unit was operated at 70 peak kilovolt (kVp), 7 mA (mA), and an exposure time corresponding to the exposed area. All the radiographs were taken with XCP receptor-holding devices (Dentsply Rinn, Elgin, IL, USA) and the paralleling technique. Photostimulable phosphor **(**PSP**)** plates (Air Techniques, Melville, NY, USA) were utilized as the receptor, and were scanned with the Scan-X Intraoral scanner (Air Techniques) after exposure. The images were stored in the EHR of the School of Dentistry, displayed on a 19-in. flat panel screen (HP Development Company, Palo Alto, CA, USA) with a 1920 X 1080 pixel resolution, and observed under a dimly lit environment.

### CBCT imaging acquisition

All of the CBCT scans were taken at the Imaging Clinic of University of Texas School of Dentistry at Houston. The included scans covered maxillary and mandibular arches with a field of view (FOV) of 150 × 90 mm^2^. The scans were acquired at 90 kVp, 10 mA, 16 s and a 0.2 mm^3^ voxel size with a Kodak 9500 unit (Carestream Health, Inc., Rochester, NY, USA). CBCT images were reconstructed with Anatomage Invivo 5 software (Anatomage Inc., San Jose, CA, USA) at 1 mm thickness. All images were viewed on the same monitor and environment as the intraoral radiographs.

### Comprehensive periodontal evaluation

#### Clinical periodontal assessment

**Fig. 1:**
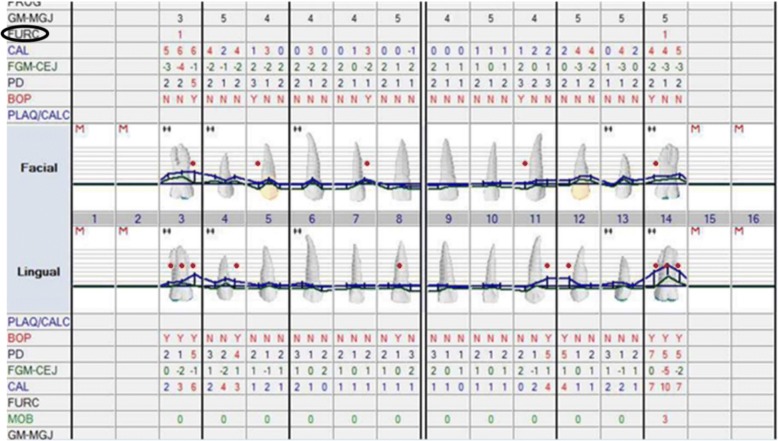
Periodontal chart demonstrates classification of molar furcation involvement

All subjects had comprehensive periodontal examination by the pre-doctoral dental students under the supervision and approval of a periodontal faculty. The evaluation included an assessment of molar furcation involvement according to modified Glickman’s classification [10] (Fig. 1). Briefly, this classification was defined as: Class I, incipient or early stage of furcation involvement, bone destruction is less than 2 mm into the furca; Class II, horizontal bone destruction extending deeper than 2 mm but less than 6 mm into the furca; Class III, horizontal bone destructions communicate between furcae of the tooth, and result in a through-and-through tunnel.

#### Intraoral radiographic assessment

**Fig. 2:**
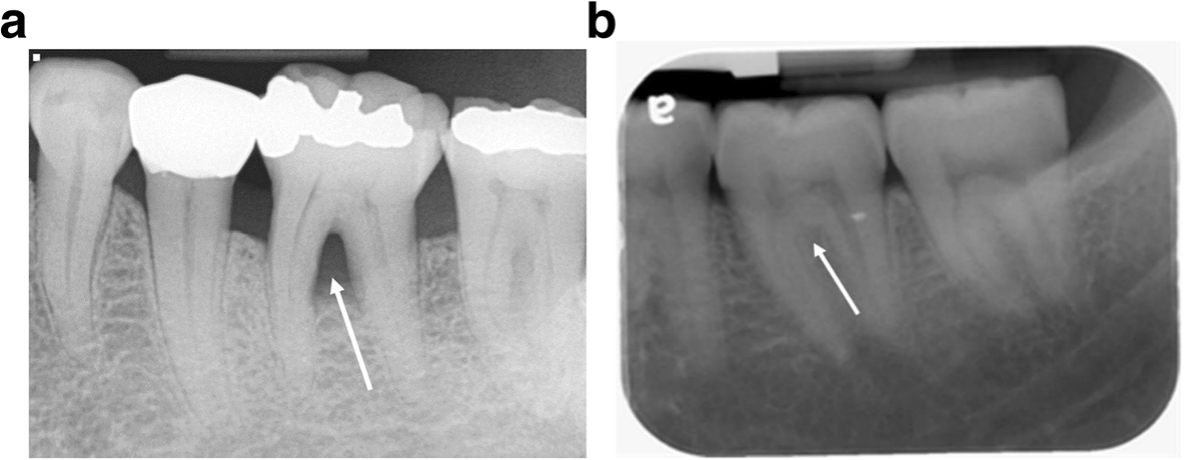
Intraoral radiographs demonstrate molar furcation status. **a** presence of furcation involvement. **b** absence of furcation involvement

First molar furcation status was evaluated on molar PA and/or BW radiographs. Presence of triangular radiolucency at the furcation area, and/or alveolar bone level was observed below furcation were radiographic signs for FI. FI was recorded as presence or absence based on the intraoral radiographs (Fig. 2).

#### CBCT imaging measurements

**Fig. 3:**
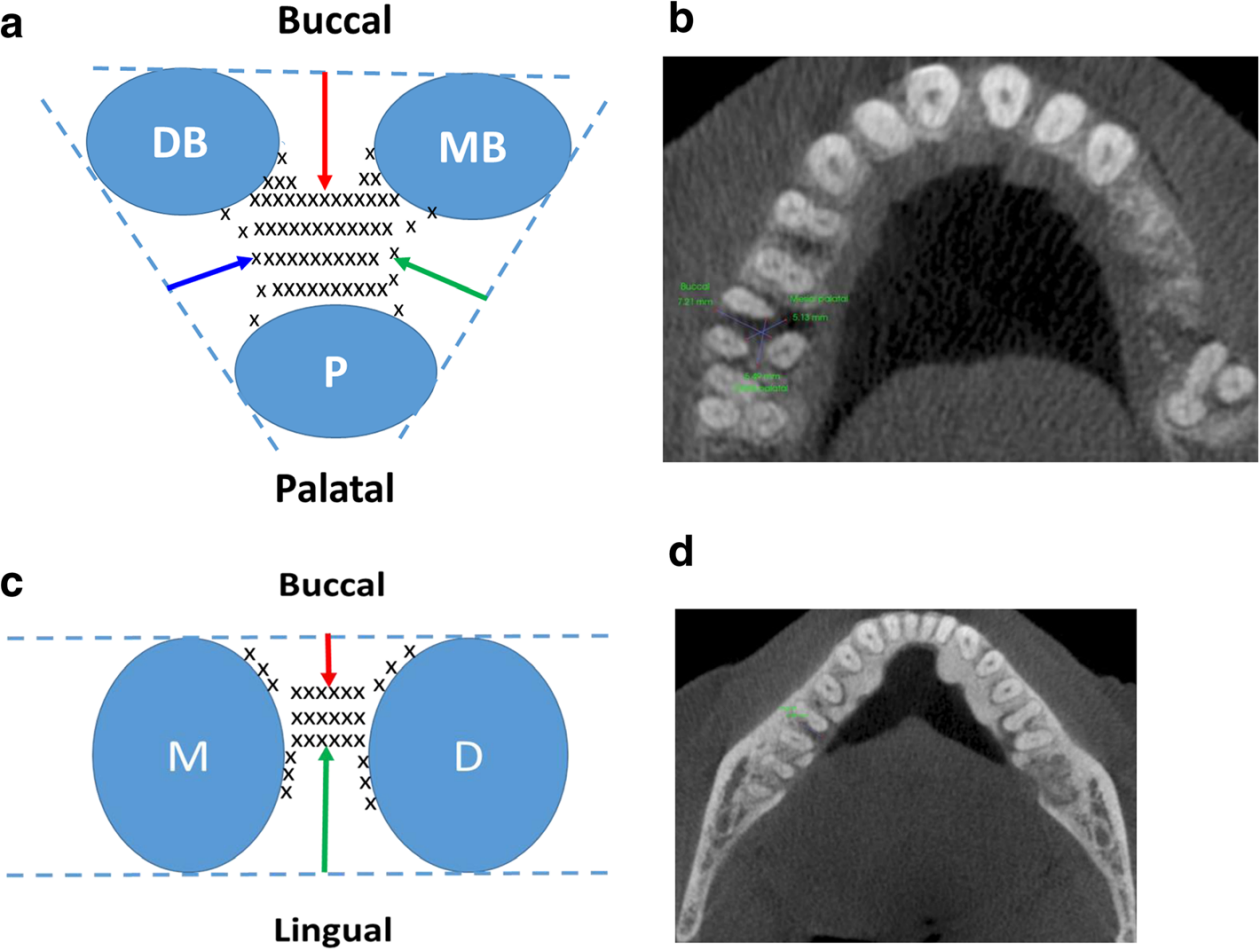
Measurement of molar furcation involvement on CBCT scans. **a** a schematic diagram illustrates measurement of furcation bone loss of a maxillary first molar. Dotted line represents tangent line connecting two adjacent root surfaces. Arrows represent distances from the middle of tangent line to the deepest point of bone loss at the different surfaces. Red, green, and blue arrows denote furcation bone loss at buccal, mesial palatal, and distal palatal surface of the molar, respectively. MB, mesial buccal root; DB, distal buccal root; and P, palatal root. **b** a representative CBCT axial view demonstrates measurements of furcation bone loss of a maxillary first molar. **c** a schematic diagram illustrates measurement of furcation bone loss of a mandibular first molar. Dotted line represents tangent line connecting buccal or lingual surfaces of the two roots, respectively. Arrows represent distances from the middle of tangent line to the deepest point of bone loss at the different surfaces. Red and green arrows denote furcation bone loss at buccal and lingual surface of the molar, respectively. M, mesial root; and D, distal root. **d** a representative CBCT axial view demonstrates measurements of furcation bone loss of a mandibular first molar

First molar furcation assessment was conducted mainly on reconstructed CBCT sagittal and axial views. Presence of FI was demonstrated as loss of trabecular bone at the furcation region on both axial and sagittal view. The depth of FI was measured on axial view where the slice showed the greatest amount of bone loss. On this slice, a line was drawn tangentially to the adjacent root surfaces. The distance from this line to the deepest point of bone loss was designated as the amount of furcation bone loss. If applicable, buccal and/or lingual furcation bone loss was measured for mandibular first molar, and buccal, mesial palatal, and distal palatal furcation bone loss were measured for maxillary first molars (Fig. 3).

All the data were analyzed by one of the co-authors KF, who was a first-year dental student and received adequate training on molar furcation assessment via intraoral radiographs and CBCT scans. The data were reanalyzed in 7 months to evaluate intra-rater reliability and reproducibility.

### Statistical analysis

Spearman’s correlation analysis was used to determine the correlations between clinical detection and intraoral radiography, clinical detection and CBCT, as well as intraoral radiography and CBCT at all the measured sites. The difference in the correlation coefficients was analyzed using Steiger’s Z-test. Intra-class correlation coefficient (ICC) was calculated to assess intra-rater reliability and reproducibility. The statistical difference was set at *p* < 0.05. The statistical analysis were run with SPSS program (version 24, IBM, Armonk, NY, USA).

## Results

Based on a previous study conducted by Qiao et al. [13] who compared molar furcation assessment between clinical probing and CBCT, a power analysis was performed which demonstrated that a sample size of 51 subjects would achieve 80% power to detect the association between these two evaluation methods on a significance level of 0.05. To ensure adequate sample size, a total of 83 patients were included in the study. Among these patients, 41 were males, 42 were females, and an age range of 31–86 with a mean age of 59.03 ± 13.08 years old.

First molar FI assessed by clinical detection, BW/PA and CBCT were illustrated in Tables 1, 2 and 3, respectively. For maxillary first molar B, MP and DP FI, clinical detection demonstrated a mean modified Glickman’s classification of 0.75 ± 0.08, 0.41 ± 0.08, 0.33 ± 0.07, respectively, and CBCT assessment revealed a mean 1.55 ± 0.22, 0.58 ± 0.05, 0.67 ± 0.12 mm bone loss, respectively. For mandibular first molar B and L FI, clinical detection demonstrated a mean modified Glickman’s classification of 0.66 ± 0.10 and 0.69 ± 0.09, respectively, and CBCT assessment revealed a mean 1.52 ± 0.19 and 1.15 ± 0.18 mm bone loss, respectively (data were presented as mean ± SD). All of the three evaluation methods demonstrated more frequent FI of mandibular first molars relative to maxillary counterpart. Of maxillary first molars, both clinical detection and CBCT revealed that buccal surface was more vulnerable for FI compared to palatal side.

**Table 1:** First molar furcation involvement assessed by periodontal probing

Modified Glickman classification^a^	Maxillary first molar				Mandibular first molar		
	B	MP	DP	Average	B	L	Average
Not present^b^	49.1%	78.4%	83.6%	70.4%	60.8%	53.9%	57.4%
Class I	34.5%	7.8%	5.2%	15.8%	18.6%	27.5%	23.1%
Class II	9.5%	6.9%	6.0%	7.5%	13.7%	11.8%	12.8%
Class III	6.9%	6.9%	5.2%	6.3%	6.9%	5.9%	6.4%
Total	100%	100%	100%	100%	100%	100%	100%

Data are presented as percentage of assayed surfaces without or with furcation involvement of corresponding category based on periodontal charting

*Abbreviations*: *B* buccal, *MP* mesial palatal, *DP* distal palatal, *L* lingual

^a^Modified Glickman classification: Class I, incipient or early stage of furcation involvement, bone destruction is less than 2 mm into the furca; Class II, horizontal bone destruction extending deeper than 2 mm but less than 6 mm into the furca; Class III, horizontal bone destructions communicate between furcae of the tooth, and result in a through-and-through tunnel

^b^Not present: no furcation involvement

**Table 2:** First molar furcation involvement assessed by periapical or bitewing radiographs

Radiographic assessment	Maxillary first molar	Mandibular first molar
Absence of furcation involvement	71.8%	66.0%
Presence of furcation involvement	28.2%	34%
Total	100%	100%

Data are presented as percentage of assayed first molars without or with furcation involvement based on radiographic assessment

**Table 3:** First molar furcation involvement measured by CBCT

Depth of furcation involvement (mm)	Maxillary first molar				Mandibular first molar		
	B	MP	DP	Average	B	L	Average
0.0	46.7%	81.5%	73.9%	67.4	45.9%	54.1%	50.0%
0.1–2.0	21.7%	5.4%	16.3%	14.5	15.3%	25.9%	20.6%
2.1–6.0	25.0%	13.0%	6.5%	14.8	36.5%	17.6%	27.1%
> 6.0	6.5%	0.0%	3.3%	3.3	2.4%	2.4%	2.4%
Total	100%	100%	100%	100%	100%	100%	100%

Data are presented as percentage of assayed surfaces without or with furcation involvement of corresponding category based on CBCT assessment

*Abbreviations*: *B* buccal, *MP* mesial palatal, *DP* distal palatal, *L* lingual

Comparison of first molar FI assessment between CBCT and clinical detection showed that, when CBCT demonstrated no furcation involvement, 18.7% of these cases were documented as FI on clinical detection. On the contrary, of the 26.7% cases identified as having 0.1–2.0 mm or 2.1–6.0 mm bone loss on CBCT, clinical detection showed no FI (Table 4). For comparison between intraoral radiographic evaluation and clinical detection, there were situations when no FI was detected on intraoral radiographs, 25.6% of these cases were demonstrated to have Class I-III FI by clinical detection. In addition, for 18.2% cases identified as FI on radiographs, clinical detection failed to detect any bone loss (Table 5).

**Table 4:** Cross tabulation of CBCT with periodontal probing for evaluation of furcation involvement for maxillary and mandibular first molars

Count		Periodontal probing				Total
		0	1	2	3	
CBCT (mm)	0.0	213	36	10	3	262
	0.1–2.0	40	24	7	0	71
	2.1–6.0	38	22	13	6	79
	> 6.0	1	2	3	5	11
Total		292	84	33	14	423

**Table 5:** Cross tabulation of intraoral radiograph with periodontal probing for evaluation of furcation involvement for maxillary and mandibular first molars

Count		Periodontal probing				Total
		0	1	2	3	
Intraoral radiograph	0 (absence)	258	75	13	2	352
	1 (presence)	58	17	39	28	142
Total		316	92	52	34	494

Spearman’s correlation and Steiger’s Z-test analysis demonstrated that clinical detection, BW/PA and CBCT were significantly correlated with each other in the assessment of first molar FI, with *r* values ranged between 0.230 to 0.644 (*P* < 0.05, Table 6). Compared with BW/PA, CBCT appeared to have higher correlation coefficients with clinical detection, especially at distal palatal side of maxillary first molar, which reached statistically significant difference (*p* < 0.05, Table 6). Between the two sets of measurements by the same rater, the ICC was 0.903, with 95% confidence interval of (0.858, 0.934), which demonstrated great reliability and repeatability of the evaluator.

**Table 6:** Correlation coefficients of periodontal probing with CBCT or BW/PA in assessment of furcation involvement for maxillary and mandibular first molars

Periodontal charting (Modified Glickman)	CBCT	BW/PA
Maxillary buccal	0.599^a^	0.579^a^
Maxillary mesial palatal	0.591^a^	0.499^a^
Maxillary distal palatal	0.644^a,c^	0.424^a,c^
Mandibular buccal	0.372^a^	0.362^a^
Mandibular lingual	0.264^b^	0.230^b^

*Abbreviations*: *CBCT* cone beam computed tomography, *BW/PA* bitewing/periapical radiographs

^a^Correlation is significant at *p* < 0.01, between CBCT and periodontal charting, or between BW/PA and periodontal charting

^b^Correlation is significant at *p* < 0.05, between CBCT and periodontal charting, or between BW/PA and periodontal charting

^c^CBCT demonstrated significantly stronger correlation (*p* < 0.05) with periodontal charting relative to BW/PA at assessment of distal palatal side of maxillary first molars

## Discussion

Our results demonstrated that all three FI assessment methods had significant correlations among each other. CBCT had stronger correlation to clinical detection than PA/BW, especially on distal palatal side of maxillary first molar. The results validate applicability of CBCT in FI assessment. Although all of the included patients had diagnosis of generalized moderate or severe chronic periodontitis, more than a half of them were not found to have FI based on the three evaluation methods.

When CBCT showed no furcation involvement, clinical detection identified 18.7% of cases with FI, indicating over-detection by clinical measurement. On the contrary, of the 26.7% cases demonstrated bone loss on CBCT, clinical detection showed no FI, suggesting under-detection by clinical detection. This was consistent with what was reported by Darby [12] and Walter [23], who also found over- and under-estimation of FI by clinical probing relative to CBCT analysis. It is speculated that probing angulation and force, soft tissue inflammation, and inter-radicular bone and root morphology, all contribute to variations of clinical detection.

Between intraoral radiographic examination and clinical detection, there were situations when no FI was identified on intraoral radiographs, about one quarter of these cases were demonstrated having FI by clinical detection. In addition, for 18.2% cases identified as FI on radiographs, probing failed to detect any bone loss. This observation confirmed the necessity of supplementing clinical detection with intraoral radiographs for the diagnosis of FI, which is reflective of the consensus in the literature [8, 14]. The inconsistency between these two methods could be due to measurement errors from either or both detecting techniques. Anatomic complexity, such as superimposition of palatal root at the furcation region may contribute to under-diagnosis of FI for maxillary molars on intraoral radiographs [5, 24], and sinus tract extending into furcation due to intrapulpal infection may lead to over-diagnosis of FI on intraoral radiographs [25], respectively.

The current study identified that mandibular first molars had more FI than maxillary first molars. In a study conducted in a Swede population, Svärdström [26] found that the prevalence of furcation involved molars was higher in the maxilla than in the mandible, based on clinical detection and intraoral radiographs. Hou et al. [27] concluded that the highest prevalence of FI was in the mandibular first molar in a Japanese population based on clinical detection. It appears that geographical locations, racial origins and evaluation modalities are among the factors contributing to variations of prevalence for molars with FI. Current study also found that FI was more frequently associated with and more severe at buccal side of maxillary first molars relative to palatal side, similarly as reported by Porciuncula [28].

Although considered a valuable addition in molar furcation assessment, CBCT is not without its shortcomings. Scatter, partial volume averaging and beam hardening artifacts could compromise its diagnostic quality, especially for patients with heavy metallic restorations, multiple endodontic treatment, orthodontic appliances, or implant prosthesis [29–31]. In addition, detectability of FI by CBCT depends on how sensitive it is to reveal bone loss at furcation area. Generally, demineralization may not be evident radiographically until it reaches approximately 30–40% [32]. This makes it challenge to detect and initiate early intervention for incipient FI of molars. In general, periodontal probing and intraoral radiographs should be used as routine examinations for detection of FI. For complicated cases when routine exams fail to provide adequate information for diagnosis and/or treatment planning, CBCT may be attempted with the smallest field of view possible and optimal exposure settings.

There were limitations for the study. It was a retrospective investigation, and the clinical detection was performed by different dental students under the supervision of board-certified periodontist, and the results were confirmed by the supervising faculty before being entering in the EHR. Still, inter-operator variations could contribute to inconsistence in the clinical detection. Also, in the present study, a relative old model of CBCT unit, Kodak 9500 was used, since this was the only CBCT unit in the Imaging Clinic of the school. This unit had a smallest voxel size of 200 μm. Compared to newer CBCT units with much smaller voxel size, such as 80 μm for Accuitomo [33], the much larger voxel size of current unit had limited spatial resolution, therefore, could limit the accuracy in the assessment of FI. In addition, current study only measured horizontal bone loss at the furcation area on CBCT scan, in order to correlate with clinical detection. Modified Glickman Classification was utilized in clinical detection, which only recorded horizontal furcation involvement of the molars. Future study could consider incorporating vertical bone loss measurement on CBCT, to gain better appreciation on furcation status. Intra-surgical FI assessment (gold standard) could be implemented, if possible, to further evaluate the accuracy of CBCT in the diagnosis of FI.

## Conclusions

CBCT has been validated as a valuable supplemental tool for assessment of molar FI in addition to periodontal probing and intraoral radiographic examinations.

## Abbreviations

BW: Bitewing
CBCT: Cone beam computed tomography
EHR: Electronic Health Record
FI: Furcation involvement
FOV: Field of view
IRB: Institutional Review Board
kVp: Kilovolt
mA: Milliampere
PA: Periapical
PSP: Photostimulable phosphor
